# Safety of 28 days consumption of a pre-workout supplement

**DOI:** 10.1186/1550-2783-11-S1-P30

**Published:** 2014-12-01

**Authors:** Jordan M Joy, Matt M Mosman, Paul H Falcone, Chih-Yin Tai, Laura R Carson, Dylan Kimber, David Choate, Rosanne M Vogel, Chad M Hughes, Michael P Kim, Jordan R Moon

**Affiliations:** 1 MusclePharm Sports Science Institute, MusclePharm Corp., Denver, Colorado, USA; 2 Metropolitan State University of Denver, Denver, Colorado, USA; 3 Grand Valley State University, Allendale, Michigan, USA; 4 United States Sports Academy, Daphne, Alabama, USA

## Background

In recent years, the consumption of multi-ingredient supplements in the pre-exercise time period in order to obtain ergogenic benefits has become increasingly popular. Ingesting pre-workout supplement(s) (PWS) is one approach used by athletes and recreational populations to aid performance and maximize training adaptations. Purported benefits include increases in strength, improved focus, and sustained energy during exercise. While research exists on the ergogenic benefits of PWS, less is known regarding the safety and potential side effects of chronic consumption. Therefore, the purpose of this study was to examine the safety of consuming a PWS containing caffeine, nitrates, and amino acids over a 28 day period.

## Methods

Forty-nine recreationally active males and females (27 ± 5 y, 172 ± 10 cm, 75.12 ± 16.06 kg) volunteered to participate in this study and were randomly divided into three groups. A control group received no PWS. The remaining two groups were instructed to ingest either one (G1) or two (G2) servings of the PWS containing caffeine, nitrates, and amino acids (Iron Pump™, MusclePharm Corp., Denver, CO) every day for 28 days. All groups were instructed to maintain normal dietary and exercise habits for the 28 days. Fasting blood and urine samples and hemodynamics were taken before and after the supplementation period. A repeated measures ANCOVA was used to analyze all data. Consent to publish the results was obtained from all participants.

## Results

Group by time interactions existed for carbon dioxide (CO2). Wherein, the control decreased from pre to post, and G2 increased from pre to post and to a greater extent than control and G1. Group by time interactions existed for red blood cell distribution width (RDW). Wherein, G1 decreased relative to control, and G2 increased relative to G1. Group by time interactions existed for globulin. Wherein, G1 increased relative to control, and G2 increased relative to G1. Group by time interactions were present for Sodium. Wherein, G1 increased from pre to post and relative to control. Group by time interactions existed for aspartate aminotransferase (AST). Wherein, G1 decreased from pre to post and relative to control, and G2 decreased relative to control and G1. Group by time interactions were present for alanine aminotransferase (ALT). Wherein, G1 decreased from pre to post and relative to control, and G2 decreased relative to control and G1. No other differences were observed, and all values remained within the normal clinical reference ranges.

## Conclusions

PWS containing similar ingredients appear to be safe for chronic consumption in recreationally active male and female populations when taken within recommended dosage guidelines. However, it may be contraindicated to consume a PWS on a daily basis for those with a predisposition to illnesses related to CO2, RDW, globulin, or sodium. Although, some variables displayed inconsistent results, such as RDW, which decreased in G1 and increased in G2. Additionally, AST and ALT decreased in the PWS groups, which is not a cause for concern.

